# Water Is Needed to Exist: Habitat Preferences of Tiger Beetles (Coleoptera: Cicindelidae) in a Desert Country

**DOI:** 10.3390/insects11110809

**Published:** 2020-11-17

**Authors:** Radomir Jaskuła, Mateusz Płóciennik

**Affiliations:** Department of Invertebrate Zoology & Hydrobiology, Faculty of Biology & Environmental Protection, University of Lodz, 12/16 Banacha, 90-237 Lodz, Poland; mateusz.plociennik@biol.uni.lodz.pl

**Keywords:** Coleoptera, Cicindelidae, Africa, Maghreb, Tunisia, habitat preferences, habitat specialization

## Abstract

**Simple Summary:**

Many species of tiger beetles are habitat specialists, which make them useful as bioindicators of the status and changes in the quality of the environment. Their diversity and community structure in particular habitats can point to the stability of habitat. We studied six different macro and microhabitat factors that influence the habitat preferences of four Cicindelidae species occurring in different Tunisian desert areas. This is the one of only a few such studies in Africa. The results clearly show high habitat specialization of most studied taxa, as well as importance of water reservoirs in species distribution in the Sahara Desert.

**Abstract:**

Tiger beetles are a group of predatory insects occurring mainly in diverse sandy areas, with particular species often characterised by narrow habitat preferences, which makes them both very important bioindicators for determining patterns of biodiversity and a flagship group for nature conservation. However, the precise roles of particular habitat parameters in the distribution of these beetles in desert areas are almost unknown. Habitat preferences for four tiger beetles species were analysed in Tunisia. Fifty samples from a major part of the country were collected, for which climate data, macrohabitat types. and soil parameters (soil humidity, salinity, pH, and structure) were studied. Here we show that most studied Cicindelidae were characterised by unique habitat preferences and did not co-occur with other ones, including two taxa found as habitat specialists, occurring in only one type of macrohabitat. Two other species were noted as more eurythopic and occupied 2–3 macrohabitat types, with *Lophyra flexuosa* as the most ubiquitous species not related to the measured parameters. The presence of a source of water (understood as a part of the habitat type, such as river banks and oases, or high soil humidity) was found as the most important factor in the distribution of the studied tiger beetle species. The present study is the first one focused on habitat preferences and habitat specialization of Cicindelidae fauna of Maghreb, and one of only a few in Africa.

## 1. Introduction

The family of tiger beetles (Cicindelidae Latreille, 1806) [1,2] includes more than 2840 species of small- to medium-sized beetles [3,4], with wordwide distribution, except some of the oceanic islands and polar regions [5,6]. Although these predatory beetles usually prefer various sandy habitats where both larvae and adult beetles live, most cicindelid taxa have narrow or very narrow habitat specialization, and can be found in only one or at most in a few very similar types of macrohabitats [7,8,9,10,11,12,13,14,15]. As a consequence, the family Cicindelidae has become a very important global flagship group for beetle and insect conservation, and tiger beetles are often used as biological indicators for determining both regional and global patterns of biodiversity [3,6,14,15,16,17,18,19,20,21,22,23,24,25,26,27,28,29,30]. On the other hand, only a few studies have focused on habitat prefereces and habitat specialization of tiger beetle species known from Africa [31,32,33], even if the Cicindelidae fauna of this continent includes hundreds of taxa [34,35].

Among various habitat parameters which play an important role in the Cicindelidae distribution, there are physical, chemical, and climatic components, including soil composition, moisture, temperature, and chemistry, as well as vegetation cover [36]. Moreover, as many tiger beetle species occupy the same areas as their larvae, habitats (especially the parameters of soil including its structure) must support the entire life of a species, from eggs (laid by females in soil) and larvae (building turrets in soil), to pupae (staying all the time at the bottom of the former larval turret), to adult beetles (living on the soil surface and looking for shelters under its layer) [6].

Taking into account data about the habitat preferences of Cicindelidae from different parts of the world, we aimed in the present paper to test the following hypotheses: (1) tiger beetles are characterised by a more or less narrow macrohabitat specialization in the studied area; (2) particular tiger beetle species prefer similar types of habitat in different regions of its distributional area; (3) occurrence of different tiger beetle species in particular regions/habitats of the studied desert country is correlated with the parametres of soil, particulary its humidity and structure.

## 2. Study Area, Material, and Methods

### 2.1. Study Area and Field Sampling

Located on the shores of the Mediterranean Sea, Tunisia is a part of the most important world biodiversity hot spot [37]. The country can be characterised by dynamic climate zones. The humid region is located in the northwest part of Tunisia and covers ca. 3.6% of the country’s total land area. The annual average rainfall in this region is 1500 mm, and the area has a typical Mediterranean climate. The semiarid area is located in the central part of Tunisia, and can be characterised by a steppe climate that accounts for 20% of the nation’s total land area. The southern area is divided into two climate regions, namely arid and desert areas. The total annual average rainfall in the arid and desert areas are 200 mm and 50 mm, respectively [38].

**Figure 1:**
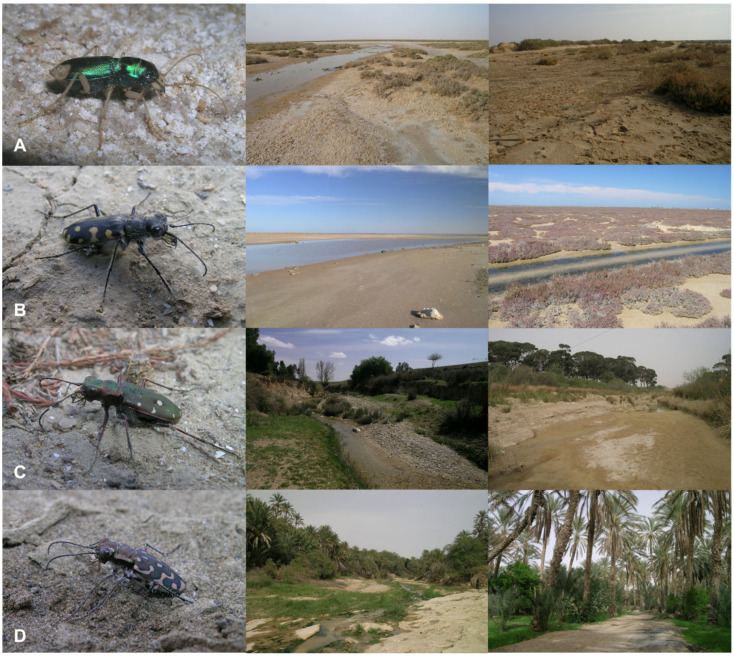
Pictures of the studied tiger beetle species and sample macrohabitats occupied by these species in Tunisia. (**A**) *Grammognatha euphratica euphratica*, (**B**) *Calomera lunulata*, (**C**) *Cicindela campestris atlantis*, and (**D**) *Lophyra flexuosa flexuosa* (photo by R. Jaskuła).

Adult tiger beetle species were collected by entomological hand nets during the TB-Quest Expedition organised to Tunisia in March–April 2010. In total, 50 samples were collected, for which location, GPS coordinates, and date were noted (Table 1). Four tiger beetle species were collected: *Calomera lunulata* (Fabricius, 1781), *Cicindela campestris atlantis* (Mandl, 1944), *Lophyra flexuosa flexuosa* (Fabricius, 1787), and *Grammognatha euphratica euphratica* (Latreille and Dejean, 1822). The material was collected during sunny hours, when the activity of most tiger beetle species is the highest, with the exception of *Grammognatha euphratica*, which is a nocturnally active beetle. This species was collected at night using the light of flashlights.Sampling sites were in recognized bioclimatic zones [39]. Every tiger beetle habitat was classified in one of the following macrohabitat types, distinguished earlier by Jaskuła [15]: banks of rivers, salt marshes, and oases (Figure 1).

Moreover, at every sampling site, soil pH and soil humidity (%) were measured in three places where tiger beetles were observed, and the average values of those measurements were noted. Additionally, three sub-samples of soil for furthern laboratory analysis were collected (in total, 150 mL of volume) in the same places of the sampling site where pH and humidity of soil were measured. Habitats were documented in photographs, too.

### 2.2. Laboratory Analysis

To check soil salinity, in the case of every sample, a volume of 2 mL of soil was soluted in 100 mL of destilated water. Then, using the WTW Multi 350i probe, electrical conductivity of the soil–water solution was measured (three measurements were done to note the average value used in further analysis).

To check the soil structure, all samples were dried separately on Petri dishes in an electronic drier. Next, every sample was weighed, and after that it was sifted on electronic sieves. All received parts of the soil particles (gravel (>2 mm), sand (0.063–2.000 mm), silt (0.063 mm–0.002 mm), and clay (≤0.002 mm)) were weighed. To estimate the proportion of particular soil particles in each sample, the values of their weight were compared with the total weight of the entire soil sample.

### 2.3. Statistcal Methods

Statistical analysis concerns 50 samples of the four Cicindelidae species: *Calomera lunulata* (*Cl*), *Lophyra flexuosa flexuosa* (*Lff*), *Grammognatha euphratica euphratica* (*Gee*), and *Cicindela campestris atlantis* (*Cca*). Insect material was supplemented by data on (1) five microhabitat environmental parameters: altitude, soil pH, soil humidity, soil salinity, and soil sediment granulometry (percentage share of gravel, sand, silt, and clay); (2) three macrohabitats: saltmarshes, river banks, and oases; and (3) six bioclimatic zones: humid, subhumid, semi-arid with mild winters, arid with mild winters, arid with cold winters, and desert. Multivariate statistics were calculated for biotic and environmental data. Principal component analysis (PCA) was conducted for environmental microhabitat ordination of sites investigated. Tiger beetle assemblages were divided into four types (A, B, C, and D) according to domination of each species. For this propose, a group-average cluster of Jaccard similarity between the presence–absence of transformed sample data (not illustrated) was used. Taxa characteristic for A–D communities and the significance of differences between those four types were obtained using similarity percentage (SIMPER) analysis with Bray–Curtis similarity. To recognise a pattern in the co-ocurrence of the investigated beetle species, non-metric multidimensional scaling (NMDS) was performed, with 50 restarts with between-species Jaccard similarity. Detrended correspondence analysis (DCA) was implemented to recognise data distribution (linear or unimodal). As the length of DCA gradient was above 3 SD units for the first two axes, canonical correspondence analysis (CCA) was conducted to recognise the main environmental factors determining species occurrence in two variants: A, with only microhabitat environmental variables included; and B, with only macrohabitats and bioclimatic zones included. CCA was conducted on presence–absence data, downweighting rare species, inter-species distance, and biplot-scaling. To test the significance of the environment–species relationship, the unrestricted Monte Carlo permutation test was applied under a full model for all environmental variables in two variants: A = microhabitat variables only, and B = macrohabitats and bioclimatic zones only. Statistical analysis was performed using PRIMER 6 and Canoco 4.5 software [40,41].

## 3. Results

### 3.1. Macrohabitat Preferences

**Figure 2:**
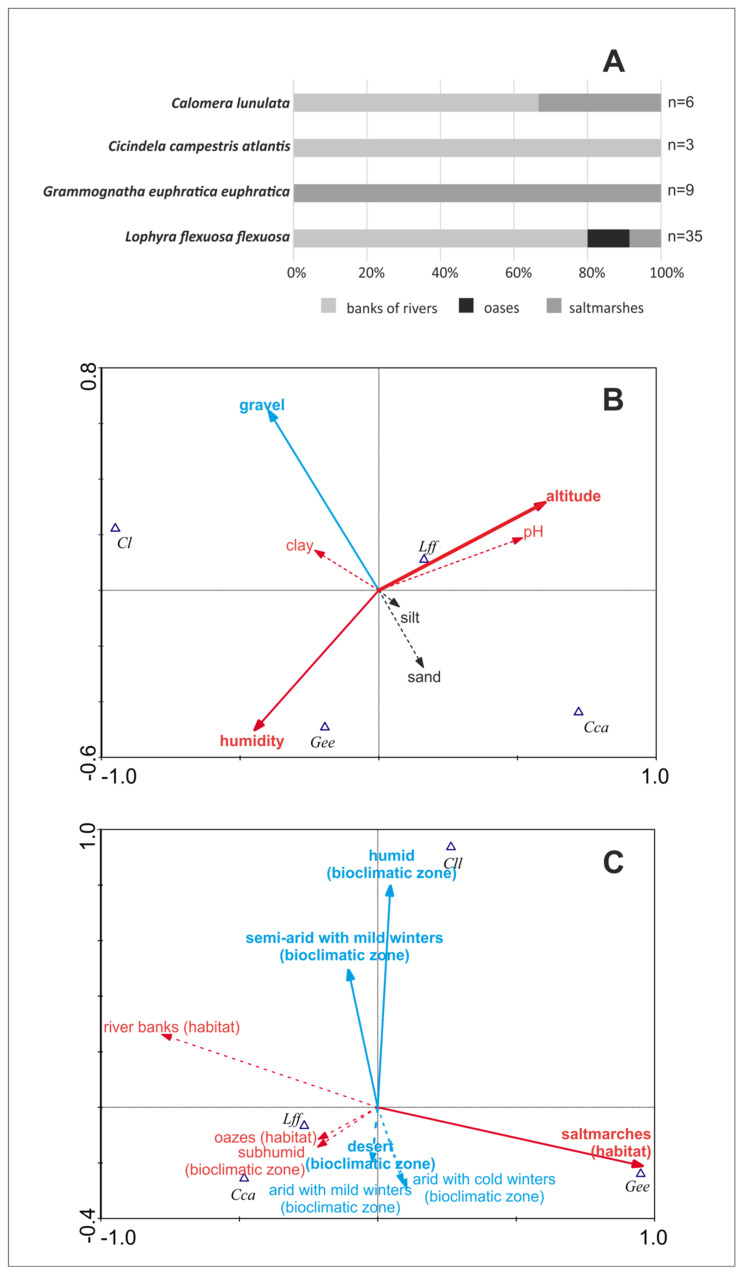
Habitat preferences of the studied tiger beetles in Tunisia. (**A**) Macrohabitat preferences. (**B**) Results of canonical component analysis (CCA) with microhabitat parameters measured as environmental variables. (**C**) Results of canonical component analysis with macrohabitat types and bioclimatic zones as environmental variables. Solid arrows (**B**, **C**) indicate environmental variables significantly explaining species variance; dashed arrows indicate insignificant environmental variables. Red arrows indicate variables correlated with axis (Ax) 1; blue arrows indicate variables correlated with Ax 2, black arrows—variables not correlated with Ax 1 or Ax 2. Gee: *Grammognatha euphratica euphratica*, Cl: *Calomera lunulata*, Cca: *Cicindela campestris atlantis*, Lff: *Lophyra flexuosa flexuosa.*

In the study, four species were recorded in four diferent macrohabitat types (Figure 1). Two tiger beetle species, *Cicindela campestris atlantis* and *Grammognatha euphratica*, were noted only in one type of habitat, respectively on river banks (*n* = 3) and saltmarshes (*n* = 4). *Calomera lunulata* occurred in two macrohabitats, including saltmarshes (33% of sites, *n* = 2) and banks of rivers (66% of sites, *n* = 4), while the most oportunistic species was *Lophyra flexuosa flexuosa*, which was found on banks of rivers (80% of sites, *n* = 28), in saltmarshes (11% of sites, *n* = 4), and in desert oases (9% of sites, *n* = 3) (Figure 2A).

### 3.2. Community Structure and Environment

**Figure 3:**
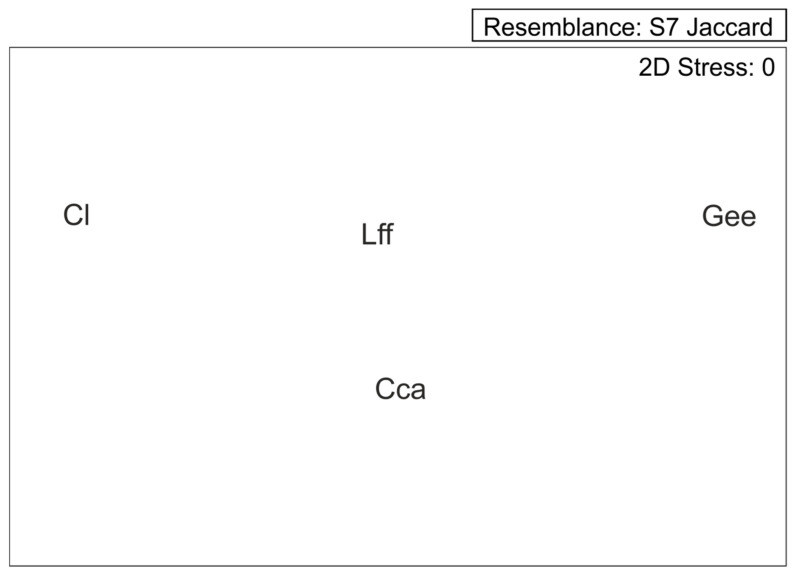
Jaccard similarity between the studied tiger beetle species based on their habitat preferences: Gee: *Grammognatha euphratica euphratica*, Cl: *Calomera lunulata*, Cca: *Cicindela campestris atlantis*, Lff: *Lophyra flexuosa flexuosa*.

**Figure 4:**
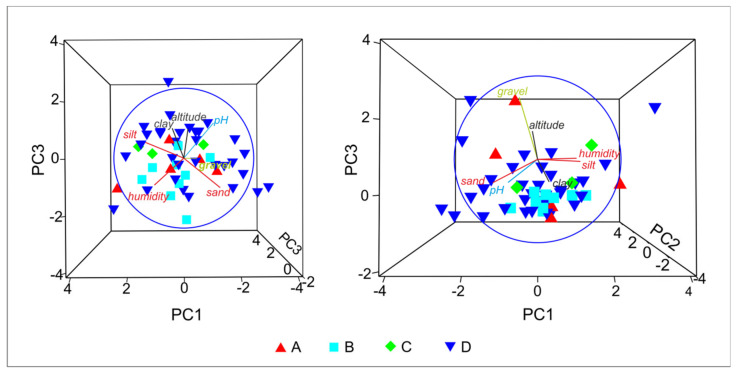
Results of principal component analysis (PCA) performed for the investigated study sites: A: sites with domination of *Calomera lunulata*, B: sites with domination of *Grammognatha euphratica euphratica*, C: sites with domination of *Cicindela campestris atlantis*, and D: sites with domination of *Lophyra flexuosa flexuosa*.

As many as four tiger beetle species were recorded in 50 samples collected in Tunisia. For this reason, the community structure is strongly simplified. All samples may be divided into four main types, according to domination of each collected species: A = *Calomera lunulata*, B = *Grammognatha euphratica*, C = *Cicindela campestris atlantis*, and D = *Lophyra flexuosa*. The MDS graph based on Jaccard similiraty (Figure 3) shows distinct differences between the ecological preferences of particular tiger beetle species, with *Lophyra flexuosa* being the most eurytopic taxon, able to co-occur with all the others. SIMPER analysis proves that they appear separately and do not tend to co-occur. PCA (Figure 4) presents environmental characteristics of the sites investigated. Sites dominated by *Calomera lunulata* (A) represent a wide variation of environmental parameters measured. Site type B, with *Grammognatha euphratica* domination, have more humid, silty sediments of lower pH and with a lower share of sand and gravel fraction. Sites of type C (*Cicindela campestris atlantis* species domination) were too few for data interpretation. Site type D (*Lophyra flexuosa* species domination) reveal a low share of gravel fraction. Nonetheless, CCA variant A (Figure 2B) partly confirms results of the PCA species–environment relation. CCA axis (Ax) 1 represents 14.4% of species variance and 56.8% of species-environment relation variance. CCA axis 2 represents 6.8% of species variance and 26.9% of species-environment relation variance. Among environmental variables, altitude (*p* = 0.01), gravel fraction share (*p* = 0.022), and soil humidity (*p* = 0.046) significantly explain species distribution among the samples (7.74%, 6.19%, and 5.03% of total variance, respectively). The share of the gravel fraction in the sediment is positively correlated with Ax 2, and the share of clay fraction and soil humidity are negatively correlated with Ax 1, whereas altitude and soil pH are positively correlated with Ax 1. The sand and silt shares in the sediment are not correlated with canonical Ax 1 or Ax 2. The results of CCA variant A indicate that *Calomera lunulata* prefers lower elevations and higher soil humidity, and *Grammognatha euphratica* and *Cicindela campestris atlantis* avoid the presence of gravel fraction in the sediment, but the other species is also an upland species present on more arid soils. *Lophyra flexuosa* seems to be a ubiquitous species not related to the parameters measured. In CCA variant B (Figure 2C), axis 1 represents 22.8% of species variance and 65.7% of species–environment relationship variance. CCA axis 2 represents 10.6% of species variance and 30.8% of species–environment relationship variance. Among environmental variables (macrohabitats and bioclimatic zones), saltmarshes (*p* = 0.02), the humid bioclimatic zone (*p* = 0.024), and a semi-arid mild winter (*p* = 0.046) significantly explain species distribution among samples, and reveal respectively 21.28%, 7.35%, and 3.87% of total variance. All three macrohabitat types and the subhumid bioclimatic zone are correlated with Ax 1, whereas the other bioclimatic zones are correlated with Ax 2. The saltmarsh habitat is positively correlated with Ax 1; the river bank and oasis habitats, as well as the subhumid bioclimatic zone, are negatively correlated with Ax 1, whereas the humid and semi-arid with mild winter bioclimatic zones are positively correlated with Ax 2. However, the desert and two arid bioclimatic zones are negatively correlated with Ax 2. Results of CCA variant B indicate that *Calomera lunulata* is distributed in the humid, semi-arid with mild winters, and arid zones; *Grammognatha euphratica* prefers saltmarshes, whereas *Cicindela campestris atlantis* and *Lophyra flexuosa* more or less avoid this kind of macrohabitat.

## 4. Discussion

The diversity and distribution of tiger beetle species is generally associated with a geographical region of the world, climate and weather conditions, as well as a habitat type (e.g., [6,18,36]). It has also been mentioned that the tropical regions’ period of year can play an important role in species distribution [31,32,33]. In regions with high average annual temperatures, high air humidity, and large mosaic of habitats—particulary different sandy areas, which are atractive for many Cicindelidae species—the diversity of this insect group can be characterised by the highest values. As a result, the number of tiger beetle species occurring in warm tropical regions is much higher that in desert areas [6,12,18,23]. Except for the climate conditions, the microhabitat structure—especially soil components, including soil composition, moisture, and chemistry—plays an important role in Cicindelidae distribution patterns. as many adult tiger beetles occupy the same areas as their ground living larvae [36]. In the case of African tiger beetle fauna, only a few papers have focused on the habitat preferences of Cicindelidae until now [31,32,33], but never with so many macro and microenvironmental factors as studied in the present paper.

The present data, even though collected in a major part of the country, allowed us to study habitat preferences of only four tiger beetle species, which make up 26% of Cicindelidae fauna known to occur in the area of Tunisia [42]. This number arose from one month of collecting in the spring period, which simply excluded the possibility of noting tiger beetle species with a different type of phenological activity (e.g., [16,43,44,45,46,47,48]). All of the studied species are more or less widely distributed in the country and the entire Maghreb region [15,42,47,48]. Although this percentage is not high, and the number of samples examined for particular species differs, our results not only clearly correspond with a lot of literature data, but also bring new, interesting data. First, the macrohabitat preferences of the studied Cicindelidae species show that most of them can be classified as habitat specialists occurring in only 1–2 habitat types. In such a group of tiger beetles, *Grammognatha euphratica* and *Cicindela campestris atlantis* (both of which were noted in only one habitat type each: saltmarshes and river banks, respectively), as well as *Calomera lunulata* (two habitat types) were found. Our present results confirm high specialisation of *Grammognatha euphratica* to only the saltmarsh macrohabitat type, as was shown earlier in the literature [15,49,50,51,52,53]. On the other hand, *Lophyra flexuosa,* the most eurythopic species in our study, noted in three different macrohabitat types, is known as a tiger beetle species characterised by one of the longest periods of activity of adult beetles among all taxa classified in this insect group in the entire Maghreb region [42,48]. It is also one of the species with the widest ecological preferences among all North African or even Mediterranean Cicindelidae, occurring in three different macrohabitat types from the sea level up to 1700 m.a.s.l. [15,52]. In the case of two other species, for which only single samples were available, we can only speculate that *Cicindela campestris atlantis* is probably a habitat specialist (known before only from two different types of macrobabitat, including one noted in our study), while *Calomera lunulata* probably can be classified as an eurythopic taxon, as our data allow us to add two more macrohabitat types to one previously known for this species in the Maghreb region [15]. Although additional data are necessary to clarify habitat preferences of some of the species studied by us, we still believe that our results show narow or very narrow habitat specialisation in the case of at least 1–2 of these species known as typical for the family Cicindelidae, not only in the Mediterranean region [14,15,54], but also in other parts of the world [7,8,9,10,11,24,45,55,56]. For example, in the United States, in Colfax County (New Mexico), only 4 of 19 species (*Cicindela fulgida*, *C. tranquebarica*, *Cicindelidia punctulata*, and *C. nigrocoerulea*) were recorded as habitat generalists, occurring in seven different macrohabitat types [45], and in the Sulphur Springs Valley (Arizona), only *Cicindelidia nigrocoerulea*, 1 of 20 species noted during the studies, was found in more than one habitat type [8]. Later, Pearson et al. [13] summarised the available data for all North American Cicindelidae, defining 17 habitat types for tiger beetle fauna, and found that only *Cicindela tranquebarica* occurred in as many as six of habitat categories. Similar results were provided also by Freitag [7] from Australia, where among 29 studied species, only two (*Myriochila mastersi* and *M. semicincta*) were found to occur in several habitat types, as well as by Acciavatti and Pearson [12] from the Indian subcontinent, where among 151 tiger beetle species, only *Calochroa flavomaculata* was recorded from several different habitat types. Additional data from Peru [9], where *Odontocheila annulicornis* was the only cicindelid taxon (of 29 species) inhabiting more than one forest habitat type in the Tambopata Reserve Zone, and from Japan [56], where only *Cicindela transbaicalica* was distributed widely along the river in the Tedori River System (two other studied species were habitat specialists), show that usually only single tiger beetle species are eurytopic.

Numerous examples from the literature show that for many epigeic tiger beetle species, soil parameters play very important roles as factors that segregate particular taxa occurring in the same macrohabitat [10,11,14,55]. Such segregation can be done, for example, on the basis of soil humidity, temperature, or soil particles, as all these components are tested at least by females after mating and before laying eggs in the soil, in order to choose the optimal microhabitat type, which can increase their reproductive success [6,26]. In warm habitats exposed to high solar radiation and high temperatures, or during the dry season in tropical regions, the presence of water reservoirs seems to be one of the key factors allowing tiger beetles to survive [6,31,32,33,36]. It seems to be especially important in regions characterised by arid or desert climates, such as Tunisia, where only about 4% of the country’s area is under the influence of a humid Mediterranean climate [38]. In the case of Cicindelidae species recorded in our study, all sampling sites were located close or very close to different water bodies (banks of rivers, oases, saltmarshes), regardless of in which region of the country the particular site was situated and by what type of climate it was characterised. Moreover, as was shown earlier by the first author of this study for other Maghrebian species, over 85% of all Cicindelidae taxa known from North Africa occupy habitats located near different bodies of water; the rest of these species are mountainous taxa, where the climate is usually more humid [15]. Among the studied tiger beetles especially, the occurrence of *Grammognatha euphratica* was strongly positively correlated with soil humidity, which can be explained by the unique specificity of the habitat type occupied by this species (Figure 2B). In contrast to river banks and oases, where water is present for a longer time or even year-round (as a result, it is much more easily available for species living in such habitats), in the case of saltmarshes, water can usually accumulate the in soil only during rainfall, while higher air humidity can be noted also at night. Although additional data for this particular species are necessary to suport this hypothesis, it can be expected that such strong habitat specialisation observed in *G. euphratica* is possible because of the beetles’ night activity. As has been shown in other Cicindelidae species by Pearson and Lederhouse [57], night activity can significantly protect tiger beetles against high temepratures and a deficit of water in the occupied habitat. On the other hand, such narrow specialisation in Cicindelidae can be explained not only by behavioural [57,58,59], but also physiological [11,60] and morphological adaptations [11,28,55,56,61], not only in adults, but also in larvae.

## 5. Conclusions

Habitat specialisation quantified by us with these Tunisian Cicindelidae species clearly reinforces suggestions of high sensitivity of this beetle group to potential environmental changes and the role of tiger beetles as important bioindicators. For these reasons, futher detailed studies of habitat/microhabitat preferences of other Maghebian Cicindelidae should be planned, not only because of a significant, direct human impact observed in most areas/habitats occupied by these insects in North Africa, but also because of a negative influence of global warming, which can cause destructive changes in water resources crucial for tiger beetles occurring in desert areas.

## Figures and Tables

**Table 1:** Sampling sites in Tunisia.

Sample Number	GPS Coordinates	Date	Sample Number	GPS Coordinates	Date
TN-01	N35.82274 E10.16047	17 March 2010	TN-23	N34.45056 E9.91836	26 March 2010
TN-02	N34.10833 E9.98197	18 March 2010	TN-24	N34.4419 E10.27603	27 March 2010
TN-03	N33.94027 E10.02673	18 March 2010	TN-25	N34.95026 E10.40299	27 March 2010
TN-04	N33.87235 E10.09325	18 March 2010	TN-26	N35.12525 E10.44033	27 March 2010
TN-05	N33.82404 E10.13745	18 March 2010	TN-27 A and B	N35.16335 E10.72168	27 March 2010
TN-06	N33.74928 E10.20916	18 March 2010	TN-28	N35.43618 E10.57555	28 April 2010
TN-07	N33.88635 E10.94381	19 March 2010	TN-29 A and B	N35.67969 E10.16462	29 April 2010
TN-08	N33.88635 E10.94381	19 March 2010	TN-30	N36.00233 E10.03985	30 April 2010
TN-09	N33.72425 E10.95342	19 March 2010	TN-31	N36.02690 E9.42404	31 April 2010
TN-10	N33.29514 E11.11316	19 March 2010	TN-32	N36.04641 E9.30721	31 April 2010
TN-11	N32.98260 E9.63695	21 March 2010	TN-33 A and B	N35.87081 E9.21404	31 April 2010
TN-12	N33.47888 E8.77212	22 March 2010	TN-34 A and B	N35.68057 E8.93391	31 April 2010
TN-13	N33.71373 E8.92086	22 March 2010	TN-35	N35.68057 E8.93391	1 April 2010
TN-14	N33.91540 E8.13387	23 March 2010	TN-36	N36.11506 E8.50126	1 April 2010
TN-15	N33.87572 E7.88200	23 March 2010	TN-37	N36.11627 E8.64001	1 April 2010
TN-16	N33.97599 E8.04028	23 March 2010	TN-38	N36.21573 E8.6220	1 April 2010
TN-17	N34.37707 E7.91309	24 March 2010	TN-39	N36.41175 E8.55772	1 April 2010
TN-18	N34.38284 E7.93288	24 March 2010	TN-40	N36.40776 E8.75538	2 April 2010
TN-19	N34.39650 E8.83120	25 March 2010	TN-41	N36.64191 E8.70025	2 April 2010
TN-20	N35.24704 E8.75249	26 March 2010	TN-42A and B	N36.85951 E8.72154	2 April 2010
TN-21	N35.20064 E8.87771	26 March 2010	TN-43	N36.91821 E9.10691	3 April 2010
TN-22	N34.65176 E9.59818	26 March 2010	TN-44A and B	N36.64673 E9.60512	4 April 2010
